# Electroporation-based proteome sampling *ex vivo* enables the detection of brain melanoma protein signatures in a location proximate to visible tumor margins

**DOI:** 10.1371/journal.pone.0265866

**Published:** 2022-05-19

**Authors:** Ilai Genish, Batel Gabay, Angela Ruban, Yona Goldshmit, Amrita Singh, Julia Wise, Klimentiy Levkov, Avshalom Shalom, Edward Vitkin, Zohar Yakhini, Alexander Golberg

**Affiliations:** 1 School of Computer Science, Reichman University, Herzliya, Israel; 2 Porter School of Environment and Earth Sciences, Faculty of Exact Sciences, Tel Aviv University, Tel Aviv, Israel; 3 Steyer School of Health Professions, Sackler Faculty of Medicine, Tel Aviv University, Tel Aviv, Israel; 4 Plastic Surgery Department, Meir Medical Center, Kefar Sava, Israel; University of California at Berkeley, UNITED STATES

## Abstract

A major concern in tissue biopsies with a needle is missing the most lethal clone of a tumor, leading to a false negative result. This concern is well justified, since needle-based biopsies gather tissue information limited to needle size. In this work, we show that molecular harvesting with electroporation, e-biopsy, could increase the sampled tissue volume in comparison to tissue sampling by a needle alone. Suggested by numerical models of electric fields distribution, the increased sampled volume is achieved by electroporation-driven permeabilization of cellular membranes in the tissue around the sampling needle. We show that proteomic profiles, sampled by e-biopsy from the brain tissue, *ex vivo*, at 0.5mm distance outside the visible margins of mice brain melanoma metastasis, have protein patterns similar to melanoma tumor center and different from the healthy brain tissue. In addition, we show that e-biopsy probed proteome signature differentiates between melanoma tumor center and healthy brain in mice. This study suggests that e-biopsy could provide a novel tool for a minimally invasive sampling of molecules in tissue in larger volumes than achieved with traditional needle biopsies.

## Introduction

Careful determination of a presence or absence of a tumor is an ideal outcome for cancer diagnostic biopsies. A major concern in biopsies is a false negative result, which happens when a test result reports that there is no disease present when, in reality, there is a disease [1]. In addition to false-negative outcomes, inconclusive biopsy results contribute to uncertainty in the currently used diagnostic assays [2]. Studies show that a positive result—both successful biopsy and molecular characterization- appears to be a reliable indicator for the presence of high-risk disease [3, 4]. However, a negative result does not reliably rule out the presence of high-risk disease, because sampled tissue may not capture the most lethal clone of a given tumor [5–8]. Although the ratio of false-negative results is usually low, in some cases false-negative combinations with inconclusive results could reach 20%, for example in liver cancer [9]. If the diagnosis misses cancer this leads to longer and more extensive and expensive treatment [10–12].

One of the most widely used tools for tissue sampling for biopsy is a needle, used in core needle biopsies or aspiration tissue biopsies. It has been reported, for some tumors, that the biopsy false-negative ratio depends on the sampling needle size [13, 14]. Indeed, increasing the needle size from 14 gauge to 11 gauge reduced the false-negative results from 22% to 3.3% in core needle biopsies of breast cancer [15]. In addition, most current molecular diagnostics studies show that single biopsy, single-site samples are not representing and even miss major tumor subclones [7, 8, 16]. Current alternatives for cellular and molecular tumor characterization—liquid biopsy approaches—do not provide information about the organ of the tumor origin [17] nor the spatial clonal composition. Thus, reliable tissue sampling remains a critical limitation to diagnoses and personal cancer medicine [4, 5, 16, 18–20].

Although it was shown that the increase of biopsy needle diameter increases the chances to capture the tumor and reduce false-negative results [15], the diameter size increase is limited due to the increased tissue damage for large needles, which could lead to complications such as bleeding and infections [21, 22]. To address these issues, and to extend the state-of-the-art of technologies that will potentially enable precision diagnosis and therapy with minimal side effects, we proposed a novel approach to molecular sampling from tissue using electroporation [23]. Electroporation procedure is widely used in medicine, biotechnology, and the food industry to increase permeabilization of cellular membranes to various molecules either for intracellular molecules extraction or for the introduction of molecules into cells [24–27]. Previous works showed that tissue electroporation can be targeted for specific regions of tissues and organs [28–33]. We also showed *ex vivo* that electroporation-based molecular harvesting extracts proteome with tissue-specific signatures for liver [23]. In the previous work *in vitro*, we showed that electroporation enables molecular transport out of the cells up to 3.9mm around the electrode [34]. Here we demonstrate the ability to gather the *ex vivo* extracted molecules for the diagnostics.

This work tests the hypothesis that liquid sampling by electroporation (e-biopsy) with a needle located outside of visible tumor margins extracts tumor-relevant proteome signature. In particular, we show that proteomic profiles obtained by e-biopsy from the brain tissue, *ex vivo*, at 0.5mm distance outside the visible margins of melanoma metastasis in mice brain have protein pattern similar to melanoma tumor center and different from the healthy brain tissue. This novel approach to solid tumors characterization differs substantially from a needle or other excision biopsy approaches, which require tissue resection and provide information limited to the size of the needle and resection tissue. It also differs from the liquid biopsy, which only measures an average biomarkers profile of the entire organism and is not organ-specific, and thus is limited to the molecular content accessible in the patient’s circulation system.

## Methods

### Animals

All experiments were conducted according to the Guidelines for the Use of Experimental Animals of the European Community and approved by the Animal Care Committee of Tel Aviv University. All *in vivo* procedures were carried out following the ARRIVE guidelines. Five C57BL/6 10-week old male mice were used in this study. The animals were housed in cages with access to food and water ad libitum and were maintained on a 12h light/dark cycle at a room temperature of around 21°C and a relative humidity range of 30 to 70%.

### Cell line culture

We chose the RET mice melanoma cell line [35, 36], kindly donated by Dr. Carmit Levi, Tel-Aviv University, since it has been shown that the RET oncogene is mutated in human melanomas, particularly in desmoplastic melanomas which have an increased risk for brain metastasis [37]. The RET cells were double-labeled with mCherry and Luc2 (pLNT/Sffv-MCS/ccdB plasmid), sorted for a red fluorescence, and selected in culture before they were injected intracranially (i.c.) as detailed below. RET cells (5x103/2μl) were implanted in C56BL/6J mice to induce tumors and the accompanying neovascularization. The cells were cultured in RPMI-1640 supplemented with 10% FBS, Pen-Strep, and 4 mg ml-1 glucose at 37°C under a humidified atmosphere of 5% CO2–95% air. Before injection, confluent monolayers of cells were released from the tissue culture flask using 0.25% trypsin and rinsed twice with serum-free RPMI-164.

### Intracranial inoculation of RET cells and BGS treatment protocol

C57BL/6 mice were anesthetized with xylazine (10 mg/kg)/ketamine (70 mg/kg). After disinfection and incision of the skin, 2 μl of RET cells (5x10^3^/2μl) were stereotaxically implanted using the following coordinates: 0.5 mm forward from the bregma, 2.1 mm lateral, and 3.0 mm ventral from the dura as we previously described [38].

### Bioluminescence analysis of the tumor size

*In vivo* bioluminescent imaging using an IVIS Spectrum CT (Perkin Elmer) was performed in anesthetized mice injected with RET-mCherry-Luc2 cells. Imaging sessions were conducted at 2-, 7- and 14-days following tumor cell implantation. Each imaging session took between 10–20 min following D-Luciferin sodium salt injection (30 mg/ml, 100 ml, i.p; Regis Technologies), as this time frame exhibited maximal and steady intensity. The analysis was carried out using Living Image software (version 4.3.1).

### Histology

The animals were anesthetized and perfused transcardially with chilled PBS, brains were removed and fixed in Bouin fixative or 4% formalin. Samples were processed for paraffin embedding by standard procedure. Coronal brain serial sections 6 μm thick were generated with a spacing of 500 microns between series. All sections which included melanoma were used for histology. From each sectional level, four slides were generated and stained by hematoxylin and eosin (H&E)

### Pulsed electric field application for proteome extraction *ex vivo* with e-biopsy

**Fig 1:**
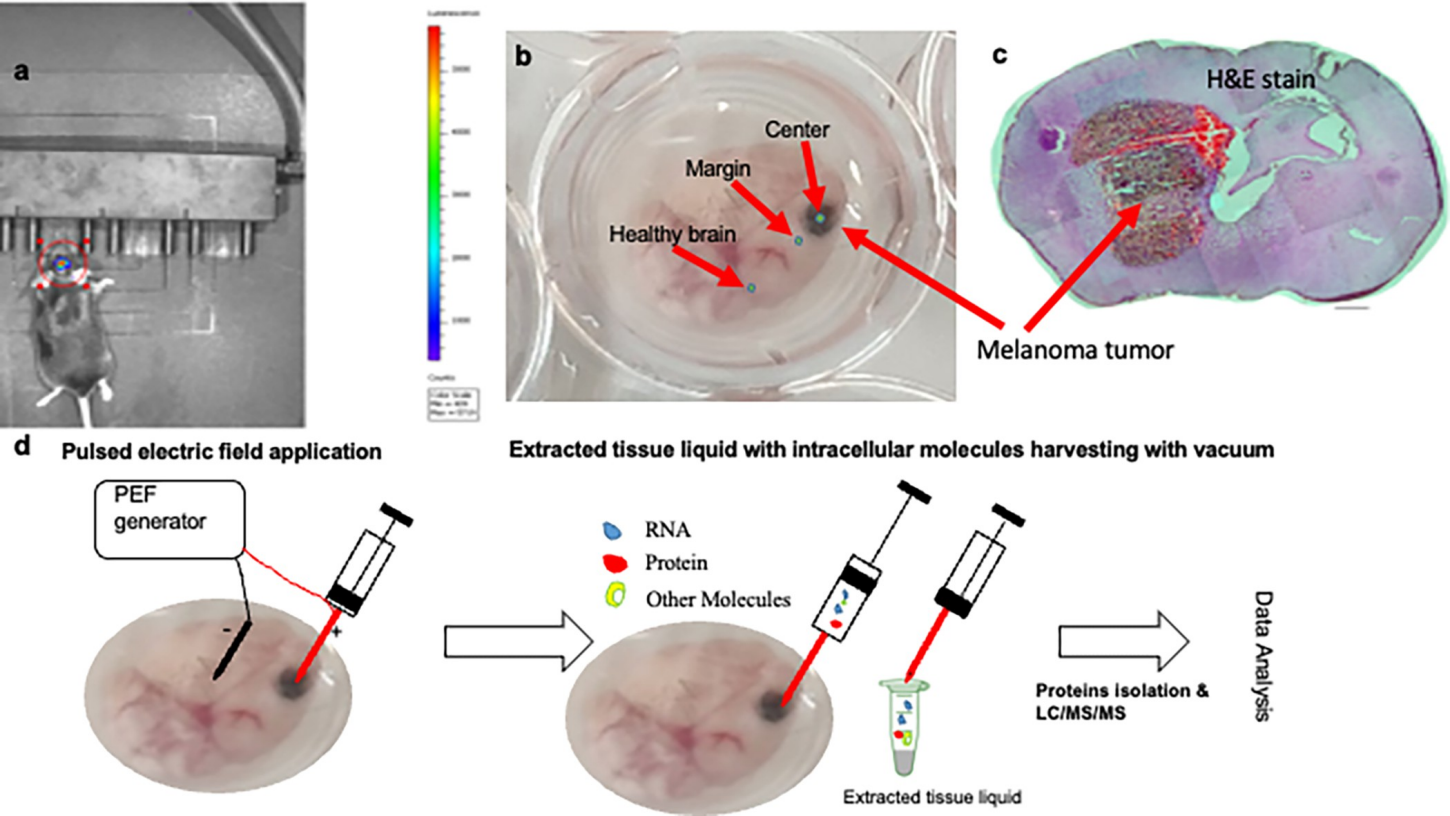
**a.** *In vivo* bioluminescent imaging of mice 14-days following RET-mCherry-Luc2 cells implantation. **b.** Excised mice brain with melanoma metastasis with labeled positions for e-biopsy **c.** hematoxylin and eosin (H&E) stain of the brain with melanoma metastasis. **d.** Schematic depiction of molecular harvesting with e-biopsy *ex vivo*.

On day 14 after the tumor cell implantation, the mice were euthanized with CO_2_. The whole brain was extracted after decapitation and out in saline. Within 2 hours after euthanization, electroporation and liquid extraction- e-biopsy-was performed in 3 positions inside the brain with a 30-G needle (**Fig 1**).

**Fig 2:**
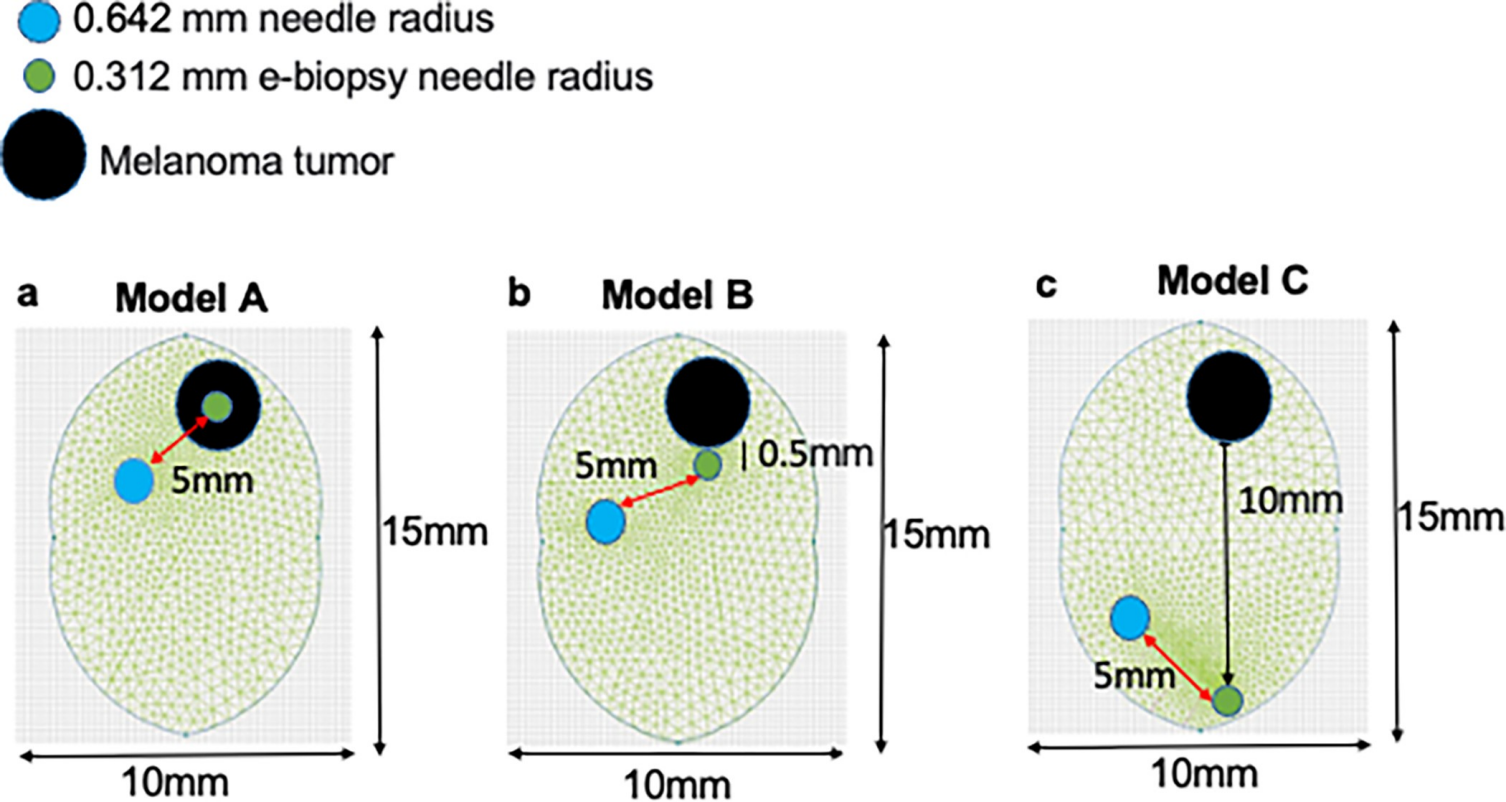
The geometry used for simulations of electric field distribution of the three experimental conditions: Center, margin, and healthy brain. **a.** e-biopsy sampling electrode is in the middle of the visible tumor (Center). **b.** e-biopsy sampling electrode is near the visible tumor margin (Margin). **c.** e-biopsy sampling electrode is far away from the tumor (Healthy brain).

To perform the procedure, the e-biopsy 30-G needle was connected to the cathode. The second, 23-G needle, connected to the anode was held at a 5mm distance from the first needle. E-biopsy was done at the center of the tumor (Center), 0.5 mm outside the visible tumor periphery (Margin), and at least 10 mm away from the tumor at the normal brain tissue (Healthy brain) (**Figs 1, 2**). The pulsed electric field was applied using our laboratory custom-made high-voltage pulsed electric field generator, described in detail in ref [39]. E-biopsy was performed using a combination of high-voltage short pulses with low-voltage long pulses [23, 40] as follows: 40 pulses, 1000 V, 40 μs, 4 Hz, and 40 pulses, 50 V, 15 ms, delivered at 4 Hz. After the application of the electric fields, the liquids were extracted from the tissue to the needle with the vacuum, manually applied with a 1.5mL syringe. The liquids were immediately transferred to 1.5 ml tubes with 100μl double distilled water.

### Proteins isolation from the e-biopsy sample

Proteins were isolated from the e-biopsy extract using the EZ- RNA II kit (Biological Industries, Beit Haemek Ltd). Homogenizing solutions were not used in the samples; phase separation solutions were directly added as follows: 0.2 ml of water-saturated phenol, and 0.045 ml of BCP. This step was followed by protein precipitation using isopropanol and wash using guanidine hydrochloride in 95% ethanol. Air-dried protein pellets were taken for proteomic analysis as described below.

### Identifying and quantifying proteins with LC-MS/MS

#### Proteolysis

The samples were brought to 8M urea, 400mM ammonium bicarbonate, 10mM DTT, vortexed, sonicated for 5’ at 90% with 10–10 cycles, and centrifuged. The protein amount was estimated using Bradford readings. 20ug protein from each sample was reduced 60°C for 30 min, modified with 37.5mM iodoacetamide in 400mM ammonium bicarbonate (in the dark, room temperature for 30 min) and digested in 2M Urea, 100mM ammonium bicarbonate with modified trypsin (Promega) at a 1:50 enzyme-to-substrate ratio, overnight at 37°C. Additional second digestion with trypsin was done for 4 hours at 37°C.

#### Mass spectrometry analysis

The tryptic peptides were desalted using C18 tips (Harvard Apparatus,MA), dried, and re-suspended in 0.1% formic acid. The peptides were resolved by reverse-phase chromatography on 0.075 X 180-mm fused silica capillaries (J&W) packed with Reprosil reversed-phase material (Dr. Maisch GmbH, Germany). The peptides were eluted with a linear 180-minute gradient of 5 to 28%, 15 minutes gradient of 28 to 95%, and 25 minutes at 95% acetonitrile with 0.1% formic acid in water at flow rates of 0.15 μl/min. Mass spectrometry was performed using Q-Exactive Plus mass spectrometer (Thermo Fischer Scientific, CA) in a positive mode using a repetitively full MS scan followed by collision-induced dissociation (HCD) of the 10 most dominant ions selected from the first MS scan.

The mass spectrometry data from all the biological repeats were analyzed using the MaxQuant software 1.5.2.8 vs. the mouse proteome from the UniProt database with 1% FDR. The data were quantified by label-free analysis using the same software, based on extracted ion currents (XICs) of peptides enabling quantitation from each LC/MS/MS run for each peptide identified in any of the experiments.

### Numerical simulations of electric fields distribution in the brain tissue and electric field-induced thermal effects

To model the distribution of the electric fields in the brain and melanoma tumor during e-biopsy, we used the finite elements method (FEM), which allows us to find an approximate solution in complex geometries for solving the Laplace differentiation equation with boundary conditions defined by the applied voltage (**Fig 2**). Numerical solutions for a Laplace equation that result in the electric field distribution in the brain and brain melanoma models were performed in QuickField (Terra Analysis, Denmark). The electric and thermal properties of tissues used to appear in **S1 Table**. The model files with full solutions appear online at the following link: https://github.com/GolbergLab/BrainEbiopsy.

We assume the thermal properties of the mice brain didn’t change after electroporation [41], while the electric conductivity after electroporation increased [42].

Direct current (DC) conduction and steady-state heat transfer problems were coupled with transient heat field problems.

In steady-state heat transfer, with Dirichlet boundary conditions, the temperature is constant with time: T_Airline_ = 25°C, where the airline differentiates between mouse brain tissue and air.

Heat sources were imported from DC conduction and steady-state heat transfer problems coupling, for the thermal field problem. To calculate the power supplied by the pulsed electric field, we used the following equation:

$$Q_{avg}=\frac{V_{RMS}^{2}}{R}=\frac{V^{2}t_{p}f}{R}$$ (1)

where *Q*_*avg*_(W) is the total average power delivered by square pulse electric field, R (ohm) is the resistance, *V*_*RMS*_ is the root mean square voltage, V (Volt) is the applied voltage, *t*_*p*_ is the duration of the pulse and *f* (Hz) is the frequency of the pulse wave.

To calculate the electric field distribution, we used the Laplace equation:

$$\nabla^{2}U=0$$ (2)

With the following potentials: $V_{Short,\;high\;voltage\;pulse}=1000V,\;V_{Long,\;low\;voltage\;pulse}=50V$, and *V*_*Ground*_ = 0.

To calculate the thermal distribution, we solved the transient heat transfer equation:

$$\frac{\partial }{\partial x}\left(\gamma_{x}\frac{\partial T}{\partial x}\right)+\frac{\partial }{\partial x}\left(\gamma_{y}\frac{\partial T}{\partial y}\right)+\frac{\partial }{\partial z}\left(\gamma_{z}\frac{\partial T}{\partial z}\right)=-q-c_{p}\frac{\partial T}{\partial t}$$ (3)

Where T is the temperature (K), *γ*(W *K*^−1^*m*^−1^) is the thermal conductivity, *c*_*p*_ (*J K*^−1^ *kg*^−1^) is the specific heat capacitance, t (s) is time, q (*Wm*^−3^) is the volume power of heat sources. In our problem q is the average volume power supplied by a pulsed electric field. We assume that heat is transferred by convection between the air, and mouse brain, and he convection coefficient with air is α = 5 W K^-1^ m^-2^ [43].

### Bioinformatics and statistical analysis

Protein intensities obtained from MaxQuant data were used for bioinformatics analysis. The analyzed dataset included 5 mice with melanoma brain metastasis sampled at three locations (**Figs 1B and 2**):

- center of the tumor (Center);
- 0.5mm from the tumor edge towards normal tissue (Margin)
- normal brain far away from the periphery (Healthy Brain).

In total, the analyzed dataset included LFQ intensities of 5072 proteins measured in 15 samples, while 4743 of them were identified in at least 3 mice and were used for further analysis. The full list of the detected protein appears online in Github: https://github.com/GolbergLab/BrainEbiopsy.

Protein intensities were binarized to “1” (LFQ-intensity > 0) and “0” (LFQ-intensity = 0) representing protein presence and absence respectively. Three binary spatial patterns (P110, P10, P001) addressing protein prevalence at a specific location (i, ii, iii) (**Table 1**) were defined.

**Table 1:** Analyzed patterns for protein detection in binary form.

Pattern	Center	Margin	Healthy Brain
P110	Protein observed	Protein observed	NO protein observed
P10	Protein observed in one or both sites		NO protein observed
P001	NO protein observed	NO protein observed	Protein observed

For each protein and each pattern of interest, the number of mice consistent with this pattern was counted. The p-value of this evaluation was defined as the probability to receive the desired pattern in at least all mice consistent with the analyzed pattern (**Eq 4**). The p-value for spatial pattern P10 was modeled as the sum of p-values for spatial patterns P110, P100, and P010.


$${p-value}_{pattern}^{protein}=\prod_{m\in M(protein,pattern)}\left[t_{Center}^{m}(pattern)∙t_{Margin}^{m}(pattern)∙t_{HealthyBrain}^{m}(pattern)\right]$$ (4)

$$s.t.:\;t_{location\;l}^{mouse\;m}(pattern)=\left\{ \begin{array}{cc} p_{l}^{m} & if\;f:\;location\;l\;is\;ON\;in\;the\;pattern\\ 1-p_{l}^{m} & if\;f:\;location\;l\;is\;OFF\;in\;the\;pattern \end{array}\right.$$

$$p_{l}^{m}=\frac{\;several\;proteins\;observed\;in\;location\;l\;in\;mouse\;m}{total\;number\;of\;proteins\equiv 4743}$$

Where *M(protein*,*pattern)* is the set of all mice consistent with the pattern for the given protein; and *t* is the probability to observe a protein in this location in the given mouse in the case when pattern requires protein existence or the complementary probability in the opposite case. The p-values for each protein-pattern pair are reported online in Github (https://github.com/GolbergLab/BrainEbiopsy) together with the corresponding FDR. The FDR to receive such p-value was calculated as Bonferroni-corrected received p-value divided by the rank of this p-value (**Eq 5**):

$$FDR\left({p-value}_{pattern}^{protein}\right)=\frac{\;expected}{observed}=\frac{4743*\;{p-value}_{pattern}^{protein}}{{rank}_{pattern}[{p-value}_{pattern}^{protein}]}$$ (5)

## Results and discussion

### Proteome harvesting in mice brains with melanoma metastasis with e-biopsy

The e-biopsy method for molecular harvesting from brain solid tumors *ex vivo* (**Fig 1A–1C**), using electroporation for cell membrane permeabilization, is shown in **Fig 1D**. First, the whole brain was isolated from the skull (**Fig 1B**). Second, the needles are inserted into the intact tissue (**Fig 1D**). Third, a series of high-voltage pulsed electric fields (PEF) are applied to permeabilize the cell membrane with electroporation. Next, a vacuum is applied on the same needle through which the PEF pulses are delivered, to pump the released cellular content into the needle and the syringe. Next, the tissue extract (~1–3μL) is discharged from the syringe to the external buffer (biology grade water), and subjected to standard protocols for molecular analysis, including purification, separation, identification (LC/MS/MS in this case), and quantification. E-biopsy can be repeated in multiple positions in the same area or other areas of the tissue sample. In our study (**Fig 1B**), excised brain with melanoma (**Fig 1C** histology) metastasis tumors were sampled 3 times each: at the center of the tumor (Center), 0.5 mm outside of the visible tumor periphery (Margin), and at least 10 mm away from the tumor at the normal brain tissue (Healthy brain).

### E-biopsy proteomics distinguishes melanoma tumor from the healthy brain and in biopsy taken 0.5 mm from the visible tumor periphery

Observed protein prevalence within and outside the tumor was analyzed according to three spatial patterns (**Table 1**): (1) proteins existing both in the tumor center and in tumor margin but not in the normal tissue (P110); (2) proteins from P110 together with those existing either in the center or in the margin (P10); and (3) proteins existing only in the normal tissue, but not in any of tumoral locations (P001). For each protein, we calculated the number of mice for which it appeared aligned to one of the patterns.

We identified 183 proteins in all 5 mice according to pattern P10. Moreover, we identified 5 proteins that have a pattern of P110 in 4 out of 5 mice, while 3 of these proteins (**Table 2**) appear in both lists: Q62167, Q3U0V1, and Q6ZWN5. Q62167 (ATP-dependent RNA helicase DDX3X), in an intracellular protein, which shuffles between the nucleolus and the cytosol [44], is strongly involved in RNA splicing and translation, and frequently dysregulated in cancers including melanoma, by targeting microphthalmia-associated transcription factor that directs a proliferative-to-metastatic phenotypic switch in melanoma^5^. We did not identify previous studies that investigated the roles of Q3U0V1 and Q6ZWN5 in brain melanoma metastasis. Previous studies linked the overexpression of Q3U0V1 (Far upstream element-binding protein 2, which also shuffles between the nucleus and the cytosol [45]) with proliferation and resistance of liver [46] and breast cancer cells [47]. Silencing of Q6ZWN5 (ribosomal protein S9, which is usually located in cell cytoplasm [48]) has inhibited the proliferation of human glioma and osteosarcoma cells [49, 50].

**Table 2:** Most abundant proteins with the pattern P110.

#	Protein Ids	Protein Names	Pattern P110		Pattern P10	
			Mice Count	P-value	Mice Count	P-value
1	P27546;A0A140T8T5;A0A0G2JFH2;A0A0G2JFT4;A0A0G2JDY5;A0A0G2JFK3;Q78TF3;A0A0G2JDU1	Microtubule-associated protein 4; Microtubule-associated protein	4	3.91E-04	4	3.12E-01
2	Q62167; P16381	ATP-dependent RNA helicase DDX3X; Putative ATP-dependent RNA helicase Pl10	4	3.91E-04	5	1.22E-01
3	A0A3B2WCD8;Q3U0V1;A0A3B2W465	Far upstream element-binding protein 2	4	2.68E-04	5	1.22E-01
4	Q60829	Protein phosphatase 1 regulatory subunit 1B	4	1.56E-04	4	1.66E-01
5	Q6ZWN5;F7CJS8;D3YWH9;Q9CXW7;D3Z673;D3YUV6	40S ribosomal protein S9; 40S ribosomal protein S9 (Fragment)	4	2.68E-04	5	1.22E-01

Interestingly, there were not many proteins that received high counts for the pattern P001. The highest count of 3 out of 5 mice was received for a single protein CON__O43790 (Keratin, type II cuticular Hb6) and only 9 received a count of 2.

### Electrode position affects the electroporation tumor coverage

The induction of cell membrane permeabilization by electroporation depends mainly on the strength of the local electric field that the cell is exposed to. To study the distributions of the electric fields in the brain tissue at different positions of the needle through which the e-biopsy is performed, we performed numerical simulations of the electric field in the three scenarios of needle positioning (**Fig 2**): Center (Model A), Margin (Model B) and Healthy brain (Model C), as described above.

**Fig 3:**
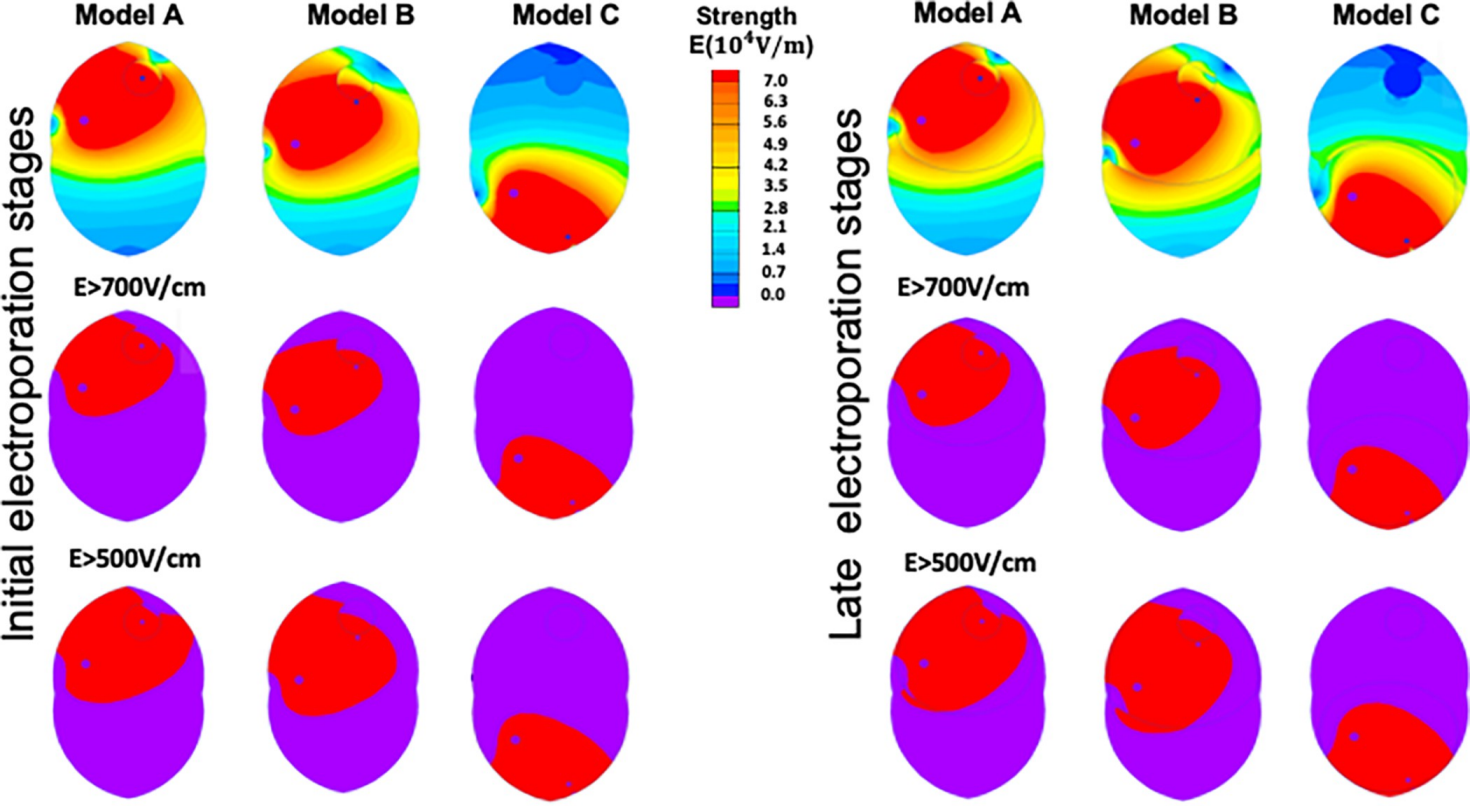
Electric field distribution in the brain with melanoma metastasis during e-biopsy. Simulations results for the three scenarios are shown: e-biopsy needle in the center of the tumor (Center, Model A), e-biopsy needle at the distance of 0.5mm from the visible tumor margin (Margin, Model B), and e-biopsy needle at the distance of 10mm (Healthy brain, Model C). The left set of models shows the electric field distribution at the early stages of electroporation: healthy brain conductivity 0.258 S m^-1^, melanoma conductivity 0.43 S m^-1^ [52]. Right panel shows the electric field distribution at the late stages of electroporation when the tissue conductivity increases [53]: brain conductivity 0.882 S m^-1^, melanoma conductivity 1.47 S m^-1^.

The simulated distribution of the electric fields in the mouse brain with melanoma metastasis for each scenario appears in **Fig 3.** As the electrical conductivity of the tissue changes during electroporation, we simulated the distribution of the electric fields in the early and late stages of the pulsed electric field protocol application. Simulation of the electric field distribution allows the detection of areas that are potentially permeabilized and, thus, from which molecular markers could be sampled. As the extent of cell permeabilization by pulsed electric fields depends on the electric field strength, we highlighted the areas of the brain that are exposed to electric fields higher than 700 V cm^-1^ and areas that are exposed to electric fields higher than 500 V cm^-1^. These threshold values have been reported in the literature as thresholds for irreversible and reversible electroporation of the brain [51]

**Fig 4:**
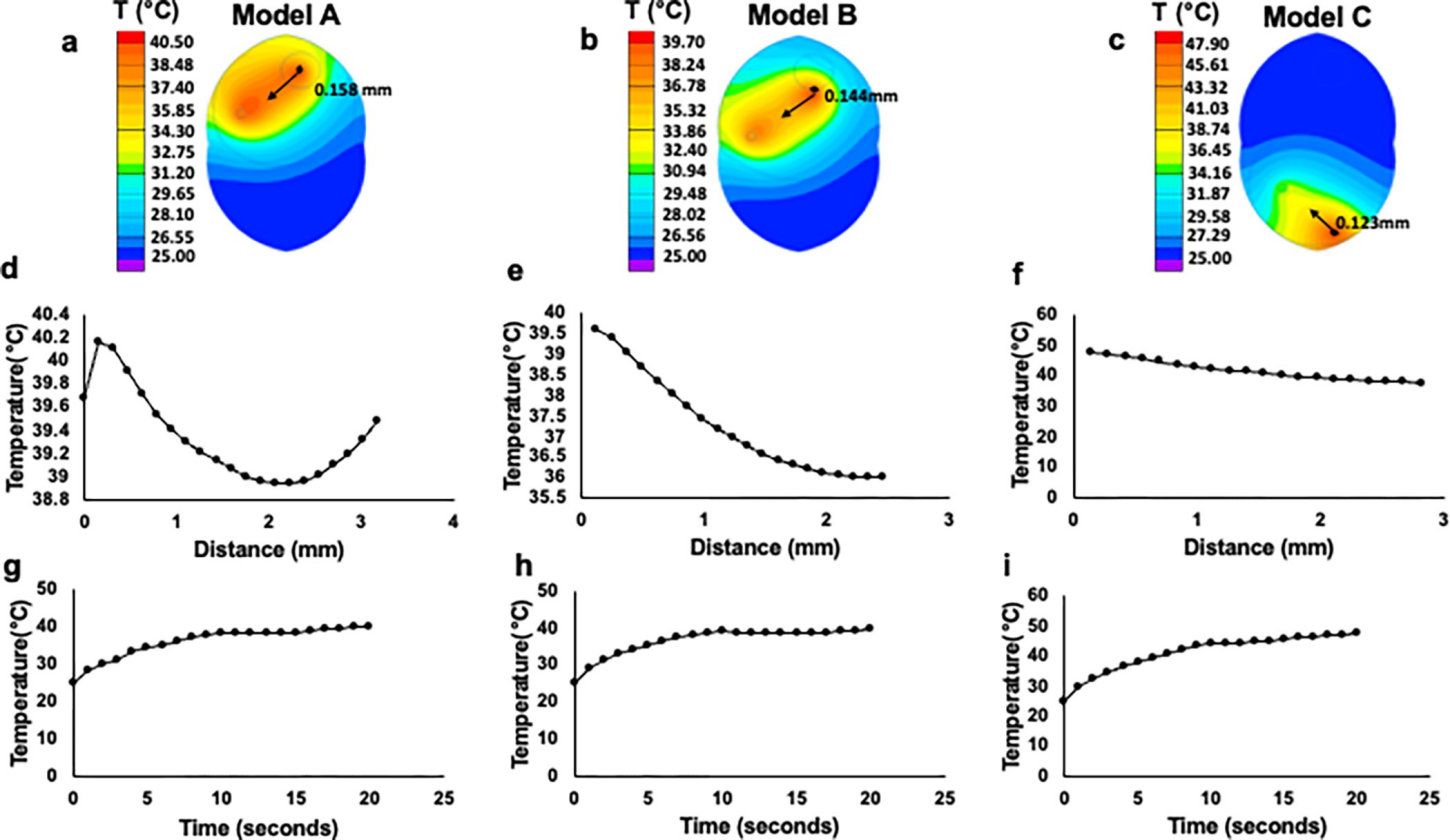
Joule heating of the brain tissue during the application of pulsed electric fields for e-biopsy. Simulations results for the three scenarios are shown: e-biopsy needle in the center of the tumor (Center, Model A), e-biopsy needle at the distance of 0.5mm from the visible tumor margin (Margin, Model B), and e-biopsy needle at the distance of 10mm (Healthy brain, Model C). Panels **a-c** show the simulation for high voltage, short-duration protocol: 40 pulses 1000 V, 40 μs, 4 Hz, coupled with the simulation for low voltage, long-duration protocol: 40 pulses 50 V, 15 ms, delivered at 4 Hz. The simulations assume electric tissue conductivities of fully electroporated tissues: brain conductivity 0.882 S m^-1^, melanoma conductivity 1.47 S m^-1^ [53]. Arrows show the contour temperature, numbers show the location of the point beneath the electrode, temperature changes in time. Panels **d-f** show temperature change as a function of distance from e-biopsy harvesting electrode over contours in panels **a-c**. Panels **g-i** show temperature change as a function of time at a point located at 0.158mm distance from the e-biopsy harvesting electrode in Model A, 0.144mm in Model B, and 0.123mm in Model C. Simulations results as presented in panels d-f give rise to the high temperatures at points that are relatively very close to the electrode, as the distance from the electrode increase the temperature decrease.

In addition, the application of electric fields on tissue could lead to heat generation by Joule heating. Both high voltage, short pulse duration, and low voltage, long pulse duration parts of the protocol are used to lead to tissue heating. The highest temperatures detected with simulation in this experiment were 40.15°C for Model A, 39.61°C for Model B, and 47.7°C for Model C. The examples of spatial and temporal changes of temperature in the tissue appear in **Fig 4.** The full solutions for the simulations for all points in time and space are available online: https://github.com/GolbergLab/BrainEbiopsy.

The performed numerical simulations are interesting, as they allow to get an initial understanding of tissues areas from which e-biopsy could potentially collect the intracellular molecular markers released by electroporation. If the electric field strength thresholds for the tissues are known, the developed simulations could indicate the fractions of tumors that are electropermeabilized. In this work, we calculated the areas of tumor covered by electric field above two thresholds E_c_: 500Vcm^-1^ and 700Vcm^-1^, which were previously reported as reversible and irreversible electroporation thresholds respectively for brain [51]. As a first approximation we calculated the visible tumor coverage% as described in **Eq 6**:

$$Visible\_Tumor\;coverage\%=100\%\frac{A_{E>E_{c}}}{A_{t}}$$ (6)

Where A_t_ is the total area of the tumor in the 2D simulation, E_c_ is the threshold of the electric field above which the tissue is electroporated, A_E>Ec_ is the area of the tumor in which the electric field is larger than the threshold electric field required for electroporation. The results that show the electroporated areas of the tumor for the three testes scenarios appears in **Table 3**.

**Table 3:** Simulated tumor sections electroporated. Simulations results for two electroporation thresholds for the three scenarios are shown: e-biopsy needle in the center of the tumor (Center, Model A), e-biopsy needle at the distance of 0.5mm from the visible tumor margin (Margin, Model B), and e-biopsy needle at the distance of 10mm (Healthy brain, Model C). The simulations assume electric tissue conductivities of fully electroporated tissues: brain conductivity 0.882 S m^-1^, melanoma conductivity 1.47 S m^-1^ [53].

	Model A	Model B	Model C
Visible_Tumor electroporated area (*mm*^2^) (E>500 V cm^-1^)	4.41±0.02	2.219±0.05	0
Visible_Tumor coverage (%) for Ec = 500 V cm^-1^	92.47	46.52	0
Visbile_Tumor electroporated area (*mm*^2^) (E>700 V cm^-1^)	3.91±0.01	1.346±0.03	0
Tumor coverage (%) for Ec = 700 V cm^-1^	82.1	28.26	0

The results shown in **Table 3** are the first estimation of the sampled by e-biopsy areas, as molecular transport from electropermeabilized cells to the harvesting e-biopsy needs depends on multiple parameters, not investigated in this work. These parameters include sampled molecules abundance in the cell, their diffusivity in the extracellular fluid and water, transport through the cell membrane, transport through the extracellular space from the cells to the harvesting needle, and transport to the e-biopsy syringe [54]. In addition, previous studies showed the indirect effect of pulsed electric fields on tissue, such as changing the environment pH [55], catalyzing multiple electrochemical reactions [56], and releasing metals from the electrodes [57], all of which could affect the sampled tissue volume and affect the sampled proteome quality. Elucidating these properties for the potential biomarkers for tumors is required for the construction of robust models of molecular transport from cells to the e-biopsy syringe. These spatial diffusion models would enable robust and repeatable molecular harvesting with electroporation in the future. Furthermore, the current numeral work was done in 2D, and further work should also incorporate 3D modeling of molecular transport in the tissue. Previous studies have reported on the 3D electric field distribution in tissues for electroporation [58], however, the next challenge is to incorporate the molecular transport to these models to make them useful for computation planning and post-treatment analysis of e-biopsy.

Thus, e-biopsy, when used in combination with *in situ* electrodes [59–61], potentially expands the opportunity for capturing molecular signature from volumes larger than electrode diameter by electroporation of larger tissue volumes around the needle. Moreover, due to its minimally invasive nature, e-biopsy potentially facilitates multiple sampling/probing, and thereby higher-resolution spatial molecular cartography of tissues at the macroscale. E-biopsy could thus enable a new type of diagnostic approach for molecular mapping of tumor and tumor environment with the potential to reduce false-negative test results and provide more precise diagnostics.

## Conclusions

In this work, we introduced a new concept for proteome probing with e-biopsy of tumor environment in proximity to the sampling needle. We showed that an e-biopsy probed proteome signature differentiates between melanoma tumor center and healthy brain in mice. Furthermore, we showed that e-biopsy probed proteome signature also potentially shows signatures that differentiate healthy brain from proximal to visual tumor margin locations, thus providing a possibility to detect tumor margins or detect tumor presence near the biopsy needle with the potential to reduce false-negative results of needle biopsies. These findings were corroborated with numerical models that showed that pulsed electric fields used for e-biopsy cover tumor areas even when the probing needle is not located inside the visible tumor tissue.

## Supporting information

S1 Table Electric and thermal properties of the mice healthy brain tissue and melanoma tissue used in numerical simulations. (DOCX)
