# Supplementary material for: Electroporation-based proteome sampling ex vivo enables the detection of brain melanoma protein signatures in a location proximate to visible tumor margins
Source: PLoS One. 2022 May 19;17(5):e0265866. doi: 10.1371/journal.pone.0265866 (PMC9119512; doi:10.1371/journal.pone.0265866)
Supplement: S1 Table — (DOCX) [file pone.0265866.s001.docx]

**S1 Table**. Electric and thermal properties of the mice healthy brain tissue and melanoma tissue used in numerical simulations.

|  | **Electric Conductivity (s/m)** | **Thermal conductivity (**$\frac{\boldsymbol{w}}{\boldsymbol{k}\boldsymbol{\cdot}\boldsymbol{m}}$**)** | **Heat Capacitance**  $\boldsymbol{c}_{\boldsymbol{p}}$ **(**$\frac{\boldsymbol{J}}{\boldsymbol{kg}\boldsymbol{\cdot}\boldsymbol{K}}$**)** | **Density**  $\boldsymbol{\rho}$ **(**$\frac{\boldsymbol{kg}}{\boldsymbol{m}^{\boldsymbol{3}}}$**)** |
| --- | --- | --- | --- | --- |
| **Mouse brain before electroporation** | 0.258  [53] | 0.565  ^11^ | 3680  ^11^ | 1039  ^11^ |
| **Mouse brain after electroporation** | 0.882  [53] | 0.565  [62] | 3680  [62] | 1039  [62] |
| **Melanoma**  **before electroporation** | 0.43  [53] | 0.565  [62] | 3680  [62] | 1039  [62] |
| **Melanoma**  **after electroporation** | 1.47  [53] | 0.565  [62] | 3680  [62] | 1039  [62] |
| $\boldsymbol{V}^{\boldsymbol{+}}$ **and** $\boldsymbol{V}^{\boldsymbol{-}}$ | 14500000  [63] | 16  [63] | 466  [63] | 8000  [63] |
